# Application of Infrared Thermography in the Detection of Hoof Disease and Lameness in Cattle

**DOI:** 10.3390/ani15081086

**Published:** 2025-04-09

**Authors:** Tina Bobić, Nikola Raguž, Mihaela Oroz, Marko Oroz, Maja Gregić, Pero Mijić, David Kranjac, Boris Lukić

**Affiliations:** Faculty of Agrobiotechnical Sciences Osijek, University of Josip Juraj Strossmayer of Osijek, 31000 Osijek, Croatia; nraguz@fazos.hr (N.R.); mioroz@fazos.hr (M.O.); moroz@fazos.hr (M.O.); mgregic@fazos.hr (M.G.); pmijic@fazos.hr (P.M.); dkranjac@fazos.hr (D.K.); blukic@fazos.hr (B.L.)

**Keywords:** infrared thermography, detection, lameness prevention, hoof disease, cattle

## Abstract

The aim of this paper was to highlight the most recent findings from research that utilized infrared thermography in diagnosing hoof disease and lameness in dairy cows. Scientific references were selected from databases based on their application of infrared thermography in detecting and preventing diseases in cattle. Studies have established that measuring view, leg position, and the presence of disease or different levels of lameness significantly affect the temperature values of cow’s feet. Additionally, the timing of measurements is also important, while factors such as lactation stage, animal activity, and ambient temperature substantially impact the infrared readings. The potential of using infrared thermography has been recognized. However, further research is needed to develop optimal combinations of different diagnostic methods to improve the accuracy and efficiency of detecting hoof disease and lameness.

## Simple Summary

Lameness is a significant economic and welfare concern facing dairy farms. Recent studies have demonstrated the potential of infrared thermography for diagnosing lameness and hoof disease in dairy cows. According to the analyzed references, there is some knowledge that the presence of disease, leg position, measurement view, and ambient temperature significantly influence the temperature values of cows’ feet. While infrared thermography is promising, further research is needed to improve accuracy and efficiency in detecting hoof disease and lameness.

## 1. Introduction

Lameness remains a significant economic and welfare issue facing dairy farms today. The factors contributing to lameness are complex and include genetic effects and farm management practices. Lameness has a negative effect on dairy herd performance and farm profitability. On average, a lame cow with dermatitis digitalis costs €307.50 to €391.80 per year or €12.10 per week per lame cow [1]. The severe lameness prevalence varies significantly among research results, ranging from 5.1% in Sweden to 45% in the USA, while in Norway and Brazil severe lameness prevalence ranges from 1.8% to 21.2%, respectively [2]. The Panel on Animal Health and Animal Welfare of the European Food Safety Authority (EFSA) recommended a twice lower threshold for lameness prevalence (up to 10%), which means that according to the numbers above, dairy farms have major problems with lameness. According to Thomsen et al. (2023) [2], the large variation in reported lameness rates indicates that while some herds can manage lameness, others have serious challenges in controlling it.

There are several ways to detect lameness on farms, the most common being the visual lameness score [3,4,5]; however, other methods, such as video surveillance [6,7,8,9], different sensor systems [10,11,12,13,14,15], etc., are increasingly being applied and tested. In recent years, infrared thermography (IRT) has gained more popularity as a diagnostic tool. IRT involves the precise measurement of infrared radiation emitted by the measured body or object. Since the 1960s, infrared thermography has been effectively applied in studying diseases, thermal physiology, and population monitoring of both wild and captive mammals [16]. IRT has been extensively studied as a potential method for improving the early detection of important animal diseases. Its primary benefit is that it is a non-invasive method for monitoring radiative surface temperature, enabling the detection of a warm body against a cool background or the assessment of underlying circulation related to physiology, behavior, and disease. The major limitation of this technique is that radiative surface temperature is also influenced by external factors such as solar radiation, wetting, and evaporation.

IRT provides a promising alternative for monitoring and surveilling cattle diseases. Despite extensive investigation, there are no established guidelines for regulating IRT parameters in animal disease detection research. Automation is the logical next step towards establishing functional IRT systems; however, approaches must first be thoroughly evaluated and validated [17]. As producers are unlikely to interpret raw temperature data from any system implemented in barns, the system must provide the end user with actionable information that enables them to take appropriate measures. Cook et al. (2021) [18] suggested continuous monitoring of the environment, housing infrastructure, and animals, while incorporating this data into a model that can make instant decisions. The use of thermal cameras in farming is currently being encouraged by advancements in camera design, software development, automated animal identification, cloud computing, machine learning, and artificial intelligence. This paper aims to highlight the most recent findings from studies that have used IRT in diagnosing hoof disease and lameness in dairy cows.

## 2. Material and Methods

A literature search was carried out using the Web of Science platform (Clarivate) including the following scientific databases: Web of Science Core Collection, BIOSIS Citation Index, Current Contents Connect, and MEDLINE. Papers published between 1974 and 2025 were taken into account.

**Figure 1:**
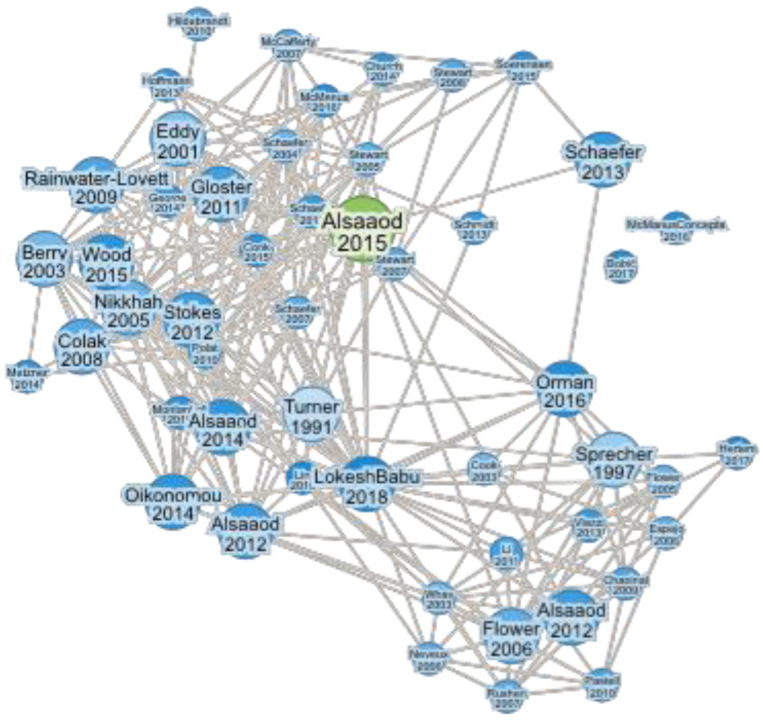
Connections between review papers according to Alsaaod et al., 2015 [19].

**Figure 2:**
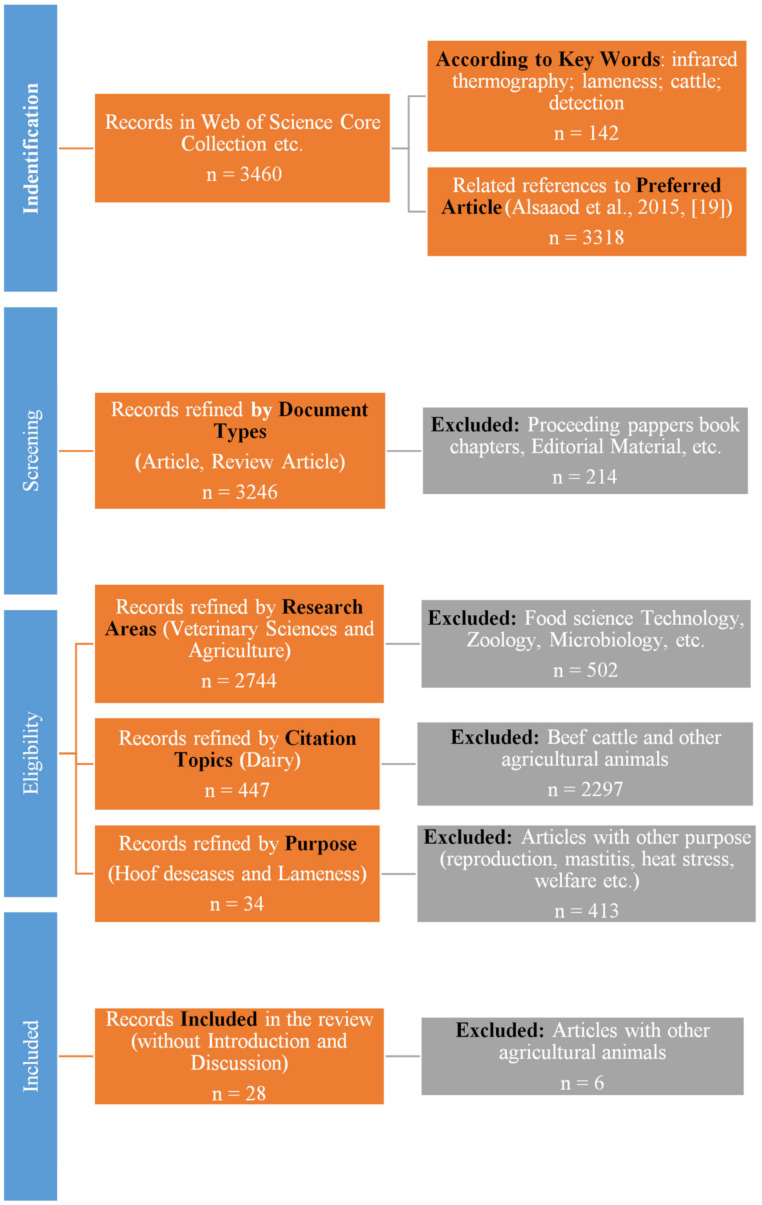
PRIZMA flow diagram.

To include any foundational articles concerning IRT methods and their use in detecting hoof diseases and lameness in dairy cattle, the following search query was employed, using English as the search language: TS = (infrared thermography* lameness* cattle* detection*). After performing the keyword search and analyzing the results, one article was singled out as representative (Alsaaod et al., 2015 [19], Figure 1), and the references related to that article were listed. Furthermore, we have narrowed down the selection regarding the type of article and chose the Article and Review Article. After that, eligibility criteria were the following: research areas (veterinary sciences, agriculture), citation topics (dairy) and purpose (hoof diseases and lameness). The selection criteria and schematic view of the literature research are outlined in the Table 1 and PRIZMA flow diagram (Figure 2).

The main research goal in this review was to identify and analyze the scientific articles that used infrared thermography in detecting hoof diseases, lameness, or prevention of lameness in dairy cows. So far, review articles have focused on the advantages and disadvantages of the application of IRT in livestock and cattle breeding, but these often lack detailed insights into specific parameter values reported in individual studies. Therefore, based on the reviewed literature, our aim was to present the most important findings in the application of IRT in lameness prevention in a clear and concise overview. We also wanted to form an impression of which characteristics were monitored and to assess the key advantages and disadvantages found in the results so far.

The aim of the literature analysis was to highlight the following key aspects:

- The main research results regarding what was measured, how it was measured, which equipment was used, and what type of data was presented;
- The main influences on the IRT readings;
- The special considerations for infrared thermography and future research.

## 3. Potential Applications and the Justification for Using Infrared Thermography

Attempts to apply infrared thermography in dairy farming date back to research from 1985 which investigated skin temperature differentials concerning estrus using the thermal infrared scanning technique [20]. A subsequent research in the 1990s examined the impact of inflammation linked to hot-iron and freeze branding [21], ear implants [22] in cattle, and the influence of micro-environmental factors on scrotal temperature [23]. In addition, some research [24] utilized a thermographic method to evaluate the management of naturally ventilated dairy barns. According to Besler et al. (2024) [25], inertial sensors (37%) and image- or video-based technologies (35%) are the most used precision technologies in cattle monitoring. However, the IRT is also quite popular (11%) compared to, for example, pressure sensors (5%), magnetometers (3%), or heart rate monitoring (2%). In scientific research, IRT was used in both small- and large-scale studies, e.g., in 42% of cases it was used in commercial or large-scale research (≥50 animals), in 20% in pilot studies (5 ≤ animals < 20), and in another 20% in medium-sized studies (20 ≤ animals < 50) [25].

Thermography can be used to identify septic arthritis in heifers [26]. By comparing thermography with radiography, the authors concluded that thermography could identify the area of inflammation. Authors emphasized that IRT provided supporting evidence for more invasive diagnostic techniques and treatment therapies, such as radiography, ultrasonography, and scintigraphy, where the localization of the site of inflammation is difficult. According to Nikkhah et al. (2005) [27], IRT also shows potential as a good diagnostic tool for laminitis, particularly in the early and mid-stage of lactation, confirming its ability to identify the effects of lactation stages and parity on hoof temperature in dairy cows. A similar finding was reported by Bobić et al., 2018 [28], indicating that the stage of lactation had a stronger effect on hoof tissue changes and, consequently, on the temperature of the coronary band, than parity. These authors determined that cows are more susceptible to diseases (78% and 88%) during the early stages of lactation (≤100 days and from 101 to 200 days) and have a higher CB temperature compared to cows in lactation for over 200 days. Other research [29] indicates that IRT is an effective technology for quickly identifying potentially infected animals during foot-and-mouth disease outbreaks. However, another study indicated that while IRT eye measurements seem to be a promising technique for determining body temperature and identifying animals with foot-and-mouth disease, their applicability to hoof temperature is limited due to the substantial impact of environmental factors and animal activity prior to IRT screening [30].

Thermographic imaging shows promise as a diagnostic tool for detecting hoof lesions in dairy cows. Combined with clinical examination, it would be a valuable technique for preventing lameness [31]. The study of Wood et al. (2015) [32] investigated the relationship between lesions and the maximum temperature of the cattle hoof to assess the significance of lesion type and changes that occurred before and after lesion treatment. Although individual lesions cannot be identified based on different temperature readings alone, this investigation demonstrated that IRT could detect the presence of a higher hoof temperature associated with foot lesions.

In contrast, Wilhelm et al. (2015) [33] found that thermography is unlikely to be suitable for the early diagnosis of subclinical laminitis in clinical settings. According to the authors, the increased temperature of the claws following calving and the obvious laminitis-like alterations (sole hemorrhages) eight weeks later did not appear to be significantly correlated.

Although the study of Lin et al. (2018) [34] was not able to achieve high levels of accuracy in predicting lameness in individual cows, it identified a high-risk group for further assessment. The authors finally concluded that IRT could serve as a tool for ranking farms according to lameness prevalence once the relevant factors have been adjusted.

Additionally, even with less expensive models, recent research has shown the potential of infrared thermo-vision cameras for lameness detection [35]. However, maximum temperature values alone were unreliable when correlating infrared measurements. While they did not directly indicate lesions, they could indicate weakened foot health [36]. While these maximal temperatures of the hoof may not directly indicate lesions, they could serve as a sign of weakened foot health that requires additional examination. Furthermore, the authors [21] proposed IRT as a potential method to help automate the monitoring of dairy herds’ foot health status.

## 4. Results of Current Applications of Infrared Thermography

### 4.1. Infrared Equipment

The effectiveness of infrared thermography in detecting specific hoof conditions or preventing lameness was highlighted in previous studies [29,30,31,35]. The results obtained by various authors reviewed here were based on similar or differing approaches and objectives. However, they all share a common goal: utilizing infrared thermography to assess hoof health and condition.

Some authors tested the equipment with lower precision and effectiveness, either due to cheaper hardware or a different primary purpose. In contrast, others used more expensive and professional thermal imaging equipment. For example, Wood et al. (2015) [32] and Lin et al. (2018) [34] used infrared thermometers, while Cockcroft et al. (2000) [26], Nikkhah et al. (2005) [27], Gloster et al. (2011) [30], Coe and Blackie (2022) [35], among others, used professional infrared cameras (Table 2). They all had the same goal: to record the surface temperature of the hooves, identify its deviations, and attempt to detect certain diseases and provide scientific evidence supporting the use of IRT in illness and lameness prevention. In the study by Coe and Blackie (2022), [35] it was concluded that low-cost IRT cameras on smartphone-based infrared thermography can be used for lameness detection. Those authors tested and compared low-cost IRT cameras with high-cost IRT cameras (FLIR) in capturing and reading the maximum environmentally adjusted temperature values of hind feet. There was no significant difference between the data captured by differing devices (sensitivity and specificity values were calculated as 66.95 and 64.53 compared with 70.34 and 70.94, respectively).

The use of infrared thermography for commercial, real-time monitoring, still involves high costs due to the need for sophisticated equipment; however, cheaper versions could also contribute to the wider use of this technology among farmers. These lower-cost options could serve as an accessible start point, allowing users to gain confidence in the method before potentially investing in more advanced systems.

However, Playà-Montmany and Tattersall [37] emphasized in their research a big influence of the spot size, distance, and emissivity, on the errors with temperature readings when working with thermal cameras. The same authors found that both spot size and distance strongly influenced the surface temperature estimates, and the angle of measurement affects the apparent emissivity of various biological surfaces (fur, feather, skin, and leaves).

### 4.2. Leg Position, Measuring Point and View

**Figure 3:**
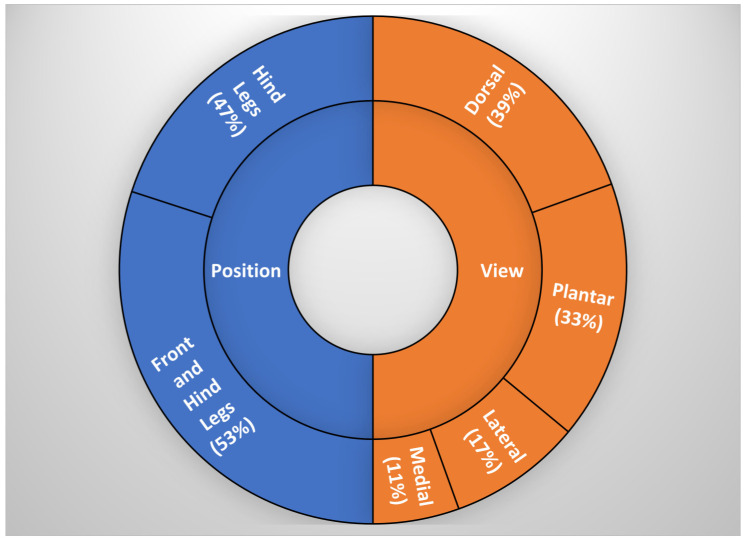
Preferred measurement views and leg positions analyzed in the reviewed studies.

**Figure 4:**
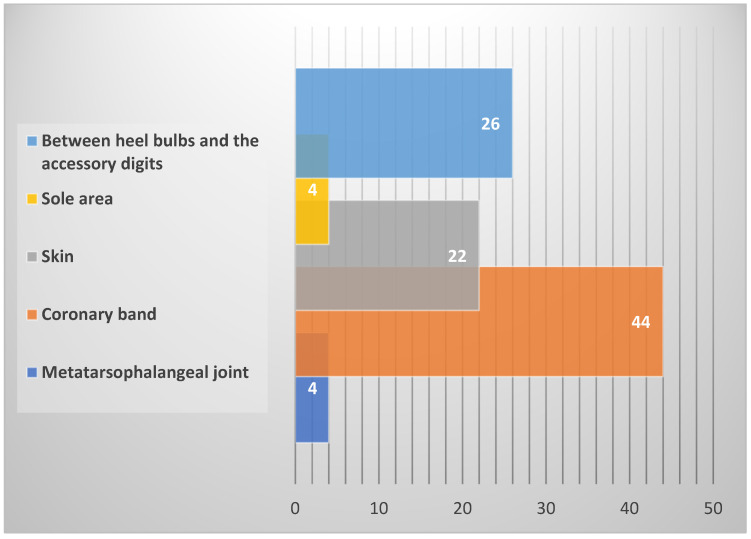
Measurement points on the cow’s feet analyzed in the reviewed studies.

Most of the studies focused on all four legs (53%; Figure 3), with a dorsal and plantar view (39%, 33%; Figure 3). Multiple spots of the feet were analyzed, with coronary band being the most extensively studied (44%; Figure 4), followed by the region between the heel bulbs and the accessory digits (26%, Figure 4), and the skin above the coronary band (22%, Figure 4).

Wilhelm et al. (2015) found a considerable temperature difference between the front and hind limbs at different weeks postpartum, with the hind limbs being noticeably warmer [33]. The medial claws tended to be warmer in the front limbs, while the lateral claws were warmer in the hind limbs.

Similar findings were reported in previously reviewed research [19], where claw temperature was measured before and after trimming. Before trimming, the lateral claws of the hind feet were significantly warmer than the medial claws. The authors associated this with the effort to balance the weight-bearing of the hind feet, which is accompanied by a measurable reduction in ΔT between the paired hind claws after routine claw trimming. A statistically significant difference in the temperature of CB was also determined between the front and rear legs [38].

The previous research [26] explained how infrared thermography can help diagnose septic arthritis in the Holstein-Friesian heifers [26]. The maximal surface temperature on the hind legs ranged from 27.2 to 31.6 °C, while on the front legs, it ranged between 21.7 and 24.8 °C. The mean range in temperature was between 21.7 and 31.6 °C (Table 3). Those temperatures were measured from the lateral, medial, plantar, and dorsal view. The affected legs showed higher temperatures on the hind legs compared to the front, while plantar measurements seem to have the lowest values regardless of whether they were taken from the front or hind legs. The authors concluded that thermography could identify the focus point of inflammation accurately.

The measurement view is important when using IRT, as significantly lower temperatures (by 1 °C; [30]) were recorded on images taken from the lateral, medial, or rear camera views compared to those taken from the front.

Significant differences in temperature values and accuracy between two measurement points (heel and coronary band) were reported in previous research [38]. At the heels of the healthy feet, the average and maximum temperature values were 24.7 °C and 30.1 °C, respectively, while at the coronary band these values were slightly higher, at 26.2 °C and 31.6 °C. Harris-Bridge et al. (2018) [39] stated that maximum temperature measured at the heels provided the highest accuracy in detecting lameness. The ROC analysis showed that AUC value of the heel ranged from 0.55 to 0.72, whereas for the CB and AUC values ranged from 0.52 to 0.67, indicating a better overall diagnostic performance of the heel as a measurement site. 

### 4.3. Measuring Values

Wilhelm et al. (2015) [33] reported a mean range in temperature for the sole area in the range from 17.12 to 19.95 °C in the first week postpartum, and from 17.12 to 20.25 °C in eighth week postpartum (Table 3). The previous research [24] explained how infrared thermography can help diagnose septic arthritis in the Holstein-Friesian heifers [24]. The maximal surface temperature on the hind legs ranged from 27.2 to 31.6 °C, while on the front legs, it ranged between 21.7 and 24.8 °C. The mean range in the temperature was between 21.7 and 31.6 °C (Table 3).

According to data presented in Table 2, different temperature values were established in previous research. In the studies that used professional infrared thermovision cameras, the lowest temperature value of the coronary band was 10.00 °C ([30]; Table 3) while the maximum temperature was 42.30 °C [29]. These values ranged from 21.2 °C to 37.3 °C in the infrared thermometer studies.

Some of the researchers presented a few cut-off values, which could be helpful in future IRT research. For instance, one research [29] identified a temperature of 34.40 °C for the coronary band, but other researchers [30] listed a lower value of 30.50 °C (75th percent certainty), and much higher values of 34.90 and 37.60 (90th and 95th percent certainty). Those values varied considerably due to the different main objectives of the experiments, shooting angles, or methods of statistical data processing. The cut-off value for CB and skin before trimming was recorded at 0.64 and 1.90 °C, and 1.09 and 2.91 °C after trimming (Table 2), according to Alsaaod and Büscher (2012) [31]. Higher cut-off values for CB were detected by Chiu and Hsu (2022), ranging from 30.10 to 34.10 °C [40] (Table 3) Furthermore, another research reported much different cut-off values for CB such as from 34.50 to 35.10 °C [41] in New Zealand and 38.10 to 38.90 °C in cows measured in Tanzania [42] (Table 3).

Meanwhile, the values for DeltaT were 0.63 °C (before trimming) and 0.73 °C (after trimming). In the region between the heel bulbs and the accessory digits, some authors [35] stated temperatures cut-off values in the amount of 1.85 and 2.40 °C, while others stated values of 23.30 and 25.25 °C [34,43]. For the regent of the cleft between the heel bulbs, the cut-off values of ≥31.0 °C were specified [36] (Table 3).

A review of the previous literature displayed certain threshold values by using various statistical models for early detection of lameness, such as 27 °C at 80% of feet with lesions [44] and 27 °C on hind legs [45]. Furthermore, Orman and Endres (2016) [46] reported specific temperature values associated with different hoof conditions: 33.5 °C and 33.7 °C for sore ulcers and 34.4 °C and 31.8 °C for general lesions, while Werema et al. (2021) [41] found that the temperature values for both feet in affected animals reached 34.5 °C.

A later study [47] based on locomotion scores (by using CART analysis) and independent variables (healthy and unhealthy (lame) animals) identified a cut-off value for maximum temperature (Tmax) at 32.40 °C. Furthermore, several studies [44,45] consistently applied a threshold value of 27 °C for maximum temperature (Tmax) in early lameness detection, i.e., to identify animals with lesions in time to prevent the development of more severe lameness within the herd.

Regarding sensitivity (SE), the authors suggested a list with the lowest values ranging from 30.0% to the highest value of 100.0%, while for specificity (SP) values ranged between 29.4% and 100.0% (Table 3). The distance from which the recording was made depended on the type of device used. The shortest distance of 0.15 cm was used for infrared thermometers, while the infrared cameras were used from a minimum distance of 0.50 to a maximum of 2.0 m.

**Table 2:** Published studies that used infrared thermography for hoof disease detection and lameness prevention in cattle—exact measurement focus.

References	Infrared Equipment	Measurement Point	Leg Position	Measurement View	Distance (m)
Cockcroft et al. (2000) [26]	IR Camera	Metatarsophalangeal joint	front and hind legs	Lateral, medial, plantar, dorsal	-
Nikkhah et al. (2005) [27]	IR Camera	Coronary band and skin	front and hind legs	Dorsal	1.50–2.00
Rainwater-Lovett et al. (2009) [29]	IR Camera × 2	Coronary band	front and hind legs	Dorsal	1.50–2.00
Gloster et al. (2011) [30]	IR Camera	Coronary band	front and hind legs	Lateral, medial, plantar, dorsal	1.00–2.00
Alsaaod and Büscher (2012) [31]	IR Camera	Coronary band and skin	hind legs	Dorsal	0.50
Wilhelm et al. (2015) [33]	IR Camera	Sole area for lateral and medial claws	front and hind legs	-	0.30
Alsaaod et al. (2015) [19]	IR Camera	Coronary band and skin	front and hind legs	Lateral, medial	0.50
Bobić et al. (2017) [38]	IR Camera	Coronary band	front and hind legs	Dorsal	1.00
Bobić et al. (2018) [28]	IR Camera	Coronary band	front and hind legs	Dorsal	1.00
Werema et al. (2021) [41]	IR Camera	Coronary band, skin, interdigital	hind legs	Dorsal	1.00
Coe and Blackie (2022) [35]	IR Camera × 2	Between the heel bulbs and the accessory digits	hind legs	Plantar	0.50
Chiu and Hsu (2022) [40]	IR Camera	Coronary band and between the heel bulbs	front and hind legs	Lateral, dorsal	1.00
Werema et al. (2023) [42]	IR Camera	Coronary band, skin, interdigital	hind legs	Dorsal	1.00
Vanhoudt et al. (2023) [36]	IR Camera	Cleft between the heel bulbs	hind legs	Plantar	0.50
Bobić et al. (2024) [48]	IR Camera	Coronary band and skin	front and hind legs	Dorsal	1.00
Feighelstein et al. (2024) [49]	IR Camera	Coronary band between the heel bulbs and the accessory digits	hind legs	Dorsal	0.35
Main et al. (2012) [43]	IR Thermometer	Between the heel bulbs and the accessory digits	hind legs	Plantar	0.15
Wood et al. (2015) [32]	IR Thermometer	Between the heel bulbs and the accessory digits	hind legs	Plantar	0.15
Lin et al. (2018) [34]	IR Thermometer	Between the heel bulbs and the accessory digits	hind legs	Plantar	0.15

IR—infrared.

**Table 3:** Published studies that used infrared thermography for hoof diseases detection and lameness prevention in cattle—exact values.

References	Measurement Point	Mean Range in Temperature (C°)	ΔT	Cut-Off Value (C°)	SE (%)	SP (%)
Cockcroft et al. (2000) [26]	Metatarsophalangeal joint	21.70–31.60	-	-	-	-
Nikkhah et al. (2005) [27]	Coronary band and skin	21.00–25.40	3.3–6.10	-	-	-
Rainwater-Lovett et al. (2009) [29]	Coronary band	23.10–42.30	-	34.40	61.1	87.7
Gloster et al. (2011) [30]	Coronary band	10.00–36.00	-	30.50 (75th percentile)	70.0 30.0	79.0 94.0
				34.90 (90th percentile)		
				37.60 (95th percentile)		
Alsaaod and Büscher (2012) [31]	Coronary band and skin	-	-	CB: 0.64 *(before trimming) CB: 1.09 (after trimming)	85.7 80.0	55.9 82.9
				S:1.90 *(before trimming) S:2.91 (after trimming)	100.0 40.0	29.4 100.0
				ΔT: 0.63 (before trimming) ΔT: 0.73 (after trimming)	85.7 93.3	35.3 68.2
Wilhelm et al. (2015) [33]	Sole area of lateral and medial claws	19.52–19.95 hind feet (1st week postpartum) 17.84–17.90 front feet (1st week postpartum) 19.45–20.25 hind feet (8th week postpartum) 17.12–17.46 front feet (8th week postpartum)	-	-	-	-
Alsaaod et al. (2015) [50]	Coronary band and skin	-	≥0.25 hind feet ≤0.13 front feet	-	-	-
Bobić et al. (2017) [38]	Coronary band	16.00–21.35 (non-lesioned feet) 18.12–27.28 (lesioned feet)	-	-	-	-
Werema et al. (2021) [41]	Coronary band, skin, interdigital	34.88–35.48	-	34.50 (for all meas. points) 35.10 (CB) 35.10 (skin above the CB) 35.10 (skin below the CB)	92.40 85.40 95.70 82.20	80.00 76.70 60.00 83.30
Coe and Blackie (2022) [35]	Between the heel bulbs and the accessory digits	18.26–26.31 (low-cost IR camera) 21.82–26.72 (high-cost IR camera	-	2.40 1.85	64.41 66.95 69.49 70.34	64.53 61.08 66.01 70.94
Chiu and Hsu (2022) [40]	Coronary band and between the heel bulbs	31.4–31.70 (non-lesioned feet, farm A) 33.8–34.10 (lesioned feet, farm A) 30.0–30.10 (non-lesioned feet, farm B) 33.5–33.70 (lesioned feet, farm B)	-	>32.05 (95th anterior, farm A) >31.45 (95th posterior, farm A) >31.25 (95th lateral, farm A) >32.05 (95th anterior, farm B) >31.45 (95th posterior, farm B) >31.25 (95th lateral, farm B)	95.45 93.18 96.02 96.41 92.31 94.87	24.00 22.67 21.33 14.29 30.36 21.43
Werema et al. (2023) [42]	Coronary band, skin, interdigital	37.02–38.29	-	38.00 (for all meas. points) 38.90 (interdigital) 38.60 (CB) 38.10 (skin above the CB) 38.70 (skin below the CB)	86.00 79.80 82.50 96.80 93.90	73.20 71.40 75.00 69.60 64.30
Vanhoudt et al. (2023) [36]	Cleft between the heel bulbs	29.7–32.1 (unwashed feet) 29.9–32.1 (washed feet)	-	≥31.0	-	-
Bobić et al. (2024) [48]	Coronary band and skin	24.5–26.6 (coronary band) 24.2–26.6 (skin)	-	-	-	-
Main et al. (2012) [43] *	Between the heel bulbs and the accessory digits	17.2–28.7 (non-lesioned feet) 21.2–37.3 (lesioned feet)	-	25.25	78	78
Wood et al. (2015) [32] *	Between the heel bulbs and the accessory digits	24.0–26.1	-	-	-	-
Lin et al. (2018) [34] *	Between the heel bulbs and the accessory digits	21.5–25.9 (non-lesioned feet) 22.2–26.9 (lesioned feet)	-	23.30	78.5	39.2

* SE—sensitivity; SP—specificity; CB—coronary band; S—skin; ΔT—difference in temperature between CB and S; IR—infrared; * authors who used infrared thermometers.

## 5. Main Influences on the Infrared Thermography Readings

### 5.1. Infrared Maximum Temperatures

Furthermore, rather than focusing on absolute temperatures, it is crucial to identify hot areas (values hotter than the surrounding skin or hotter than other feet) [28,30,31,38]. According to Vanhoudt (2023) [36], who examined the impact of the dichotomization on the infrared maximum temperatures (IRTmax), and established that IRTmax alone could probably be used for automated detection of feet with lesions in M2 stages (the presence of an ulcerative lesion ≥2 cm in diameter with a red–grey surface, developed by Döpfer et al. [51]). IRTmax is a tool that can help automate monitoring of the foot health status of dairy herds, and it most likely plays a part in detecting feet that are at risk for poor foot health and require additional examination.

### 5.2. Presence of Foot Lesions and Lameness Score

Alsaaod and Büscher (2012) [31] confirmed an increase in the surface temperature of the lame limb when a hoof had a lesion. The temperatures of coronary band (CB), skin above the coronary band (S), and ΔT (difference in temperature between CB and S) were significantly higher in cows with fewer days in lactation (≤200 days in milk vs. >200 days in milk) for all healthy hooves [27,28,31]. In an effort to understand the influence of parity and stage of lactation on the temperature values of some parts of the hoof, Bobić et al. (2018) [28] made several conclusions: Parity did not have a significant effect on the temperature of the coronary band (CB) for cows with lesions, while the stage of lactation did (*p* < 0.0001). In cows without lesions, both parity and the stage of lactation had a significant (*p* < 0.01; *p* < 0.0001) influence on CB temperature. The authors [28] indicated that the stage of lactation had a stronger effect on the tissue changes in the hooves than parity, and consequently, on the temperature of the coronary band.

The authors of the previously described study [32] were able to detect elevated foot temperature associated with foot lesions, although specific lesions did not seem to affect the temperature differently. They concluded that feet with claw horn lesions generally displayed a higher temperature than those without lesions, by around 0.393 °C. In comparison, feet with multiple lesions had a significantly higher foot temperature than feet with no lesions present, by approximately 2.005 °C. The same authors [32] reported highly significant differences in maximum, minimum, and mean measured temperature of CB on cows’ hooves between non-lesioned and lesioned hooves. The significant influence of hoof disease on temperature values was confirmed by Harris-Bridge et al. (2018) [39]. They reported statistically significant differences between lame and healthy feet at two anatomical locations (heels and CB). The lame feet had significantly higher temperatures at both the heel and CB compared to healthy feet.

The lameness stage also influences cows’ foot temperature, because there is a positive correlation between the foot temperature and mobility score [34]. Hoof temperatures of cows with a lower degree of lameness (mobility score 1) were found to be lower compared to cows with a higher degree of lameness (mobility score 2 and 3). The foot temperature of the lame cow was significantly higher than that of non-lame cows. On the contrary, Renn et al. (2014) [45] did not observe significant difference in the mean hoof temperatures across different lameness scores. However, the same authors found a significant correlation between individual foot temperatures, suggesting that while average group differences were not statistically evident, temperature readings within individual cows still showed consistent patterns.

### 5.3. Environmental Condition and Metabolic Status

The temperatures of CB and S regions are positively correlated with ambient temperature. A positive correlation of the hoof temperatures (CB, S, sole area, etc.) with ambient temperature was indicated in many other studies [19,30,31,33] as one of the major disadvantages of using IRT in detecting hoof problems and lameness. According to the prediction model established in recent research [32], for every ±1 °C change from the average ambient temperature (13.6 °C), the foot temperature will change ±0.28 °C.

In addition to ambient temperature, the ventilation, relative humidity, and direct solar radiation can also alter infrared temperature results [52,53]. These environmental factors affect heat exchange through convection and evaporation, potentially leading to increased evaporation rates with consequent decreases in temperature at the sites of sweat evaporation. This may result in lower temperature readings, particularly in areas where sweat evaporation occurs, introducing errors in the interpretation of IRT data. According to Mota-Rojas (2021) [54], the cattle’s body surface temperature fluctuates for a variety of reasons that can influence the interpretation and validation of infrared temperature readings. These include anatomical and physiological differences among large animals, as well as the impact of the environment, gender, and species. The metabolic status of the animal at the time of measuring can also affect feet temperature readings. A significant positive correlation (correlation = 0.88, *p* < 0.001) between heat production and feet temperature has been observed [55]. Previous studies on energy metabolism have demonstrated that more metabolically efficient cattle have both lower heat loss [56] and lower methane production [57]. Furthermore, Chiu and Hsu (2022) [40] confirmed that cows without hoof lesions have a significantly higher daily activity and feeding time compared to those with lesions. Variations in anatomical and physiological traits such as thermal insulation, tissue conductance, distance to blood vessels, and regional blood flow, also play a major role in determining surface temperature. Therefore, particular caution is advised when interpreting animal temperature values during the application of infrared thermography. It is crucial to perform measurements under stable ambient temperature with identical conditions and avoid extreme ambient temperatures (e.g., too hot, too cold, windy, etc.) [58].

### 5.4. Spot Size, Distance and Emissivity Errors

Playà-Montmany and Tattersall (2021) [37] emphasized that camera spot size and angle of measurement are crucial for the accurate assessment of objects. For reliable results, the camera needs to be close enough to the object with the angle as low as possible. The greater the distance between the camera and the observed object, the lower the temperature reading. This is due to the fact that as an item moves farther away, gases in the air absorb more infrared light from it. Emissivity decreases with increasing angle of incidence, which means any angle > 55° leds to a 5% decline in emissivity [37]. For this reason, it is necessary to always record animals from the same distance, ideally with the camera focused at an angle of 45 degrees) [58].

Under farm conditions, it is very difficult to approach the cattle very close, because cattle will flee if the distance is too close. In addition, you should avoid shooting too close to the cows due to the risk of spraying dung, urine, etc., which can further disrupt the camera readings. However, covering camera with a plastic bag can be some kind of a solution for protecting the camera without significantly impairing thermal readings [59].

## 6. Special Considerations for Infrared Thermography and Future Research

Based on the review and analyses of the results from the cited authors, the following key conclusions have emerged:

➢ The measuring view is important and significantly affects the temperature values of the cow’s feet. Images taken from the lateral, medial, or rear views have a temperature lower by around 1 °C than those taken from a front view.
➢ The leg position also significantly affects foot temperature; the rear legs have higher temperature values than the front legs. Additionally, temperature differences exist between the front and hind legs’ lateral and medial sides.
➢ The parity does not have a significant impact on the temperature of the coronary band for cows with lesions, but the stage of lactation has a strong influence on the tissue changes on cow’s feet.
➢ The presence of disease or some inflammation on the feet also has an impact on the temperature values of the cow’s feet. However, the determination of specific lesions cannot be identified by temperature difference.
➢ The lameness and lameness score show a positive correlation with the surface temperature of the feet.
➢ The ambient temperature positively correlates with the surface temperature of the feet.

Welfare issues on farms must not be disregarded; modernization and development are required, as well as finding ways to reduce production costs. Although the application of new technologies requires certain financial investments at the beginning, their long-term benefits (prevention of diseases and improvement of management) can lead to a significant reduction in costs and the improvement of animal welfare. According to Thomas et al. (2022) [60], IRT is one of the developing strategies for animal pain mitigation and detection, which can help increase cow welfare. These authors found a significantly increased maximum hoof temperature and pain (applying significant pressure) in cows affected by varying degrees of digital dermatitis. The application of precision technology has spread throughout the world, but the countries with the highest number of scientific papers on cattle monitoring using precision technologies were the following: China (*n*= 55), Japan (*n* = 52), the United States (*n* = 38), followed by Australia (*n* = 25), and India (*n* = 20) [25]. Future research directions may include deep learning-based computer vision techniques for early detection and prediction of hoof diseases using infrared thermography data. Recent findings indicate that the use of IRT images in conjunction with artificial intelligence-based predictors holds great potential for developing real-time automated tools to monitoring hoof diseases in dairy cows [49].

Infrared thermography is a promising method with strong potential for use in future research and practical applications in livestock monitoring. However, the challenges that accompany its application for commercial purposes are visible and impose the need to overcome their technical and methodological limitations. One way to improve the application conditions and reliability of infrared thermography is to include multiple parameters during recording and analysis of thermograms. In addition, along with ensuring controlled ambient conditions, it is also essential to individualize the data for each animal and include the animal as a fixed effect. It is also necessary to combine and use, in addition to thermal imaging, other sensors and techniques for detecting hoof diseases and preventing lameness. Further progress of the practical applications of thermal imaging is seen through the use of stationary thermal cameras that will monitor animals in real time, combined with other sensors for precision framing.

## 7. Conclusions

Recent studies have demonstrated the potential of infrared thermography for diagnosing lameness and hoof disease in dairy cows. So far, it has been established that factors such as measuring view, leg position, and the presence of disease or varying levels of lameness significantly affect the temperature readings on the cow’s feet. Additionally, the timing of measurements is crucial, as the stage of lactation, animal activity, and ambient temperature all impact infrared readings. The potential of infrared thermography is well recognized; however, further research is needed to develop optimal combinations of different methods to improve the accuracy and efficiency of detecting hoof disease and lameness.

## Figures and Tables

**Table 1:** Inclusion criteria for the literature search.

Criteria		Notes
Main objective	Studies on using infrared thermography in detection of hoof diseases, lameness, or lameness prevention	Studies that used other methods of detecting disease or lameness were excluded.
Type	Article and review article	Abstracts, conference papers, and preliminary studies were excluded.
Research area	Veterinary sciences, agriculture	Zoology, biology, physiology, endocrinology, environmental sciences, ecology, and similar areas were excluded.
Topics	Dairy farming	Beef cattle and other agricultural animals were excluded (pigs, sheep, goats, horses).
Purpose	Detection of hoof disease and lameness	Articles with other purposes were excluded (reproduction, mastitis, heat stress, welfare, etc.).

## Data Availability

No new data were created or analyzed in this study.

## Abbreviations

The following abbreviations are used in this manuscript

EFSA	Panel on Animal Health and Animal Welfare of the European Food Safety Authority
IRT	Infrared thermography
CB	Coronary band
S	Skin above the coronary band
ΔT	Difference in temperature between the coronary band and skin
IRTmax	Infrared maximum temperatures
